# Osteoporosis-related characteristics in care home residents in England: a retrospective cohort study

**DOI:** 10.3399/BJGPO.2022.0142

**Published:** 2023-04-19

**Authors:** Vaiva Gerasimaviciute, Rohini Mathur, Kathryn Elizabeth Mansfield, Matthew Paul McDermott, David Edward Neasham, James Liam O'Kelly

**Affiliations:** 1 Amgen Ltd, Uxbridge Business Park, Uxbridge, UK; 2 Center for Primary Care, Wolfson Institute of Population Health, Queen Mary University of London, London, UK; 3 London School of Hygiene and Tropical Medicine, London, UK

**Keywords:** homes for the aged, osteoporotic fractures, general practice

## Abstract

**Background:**

The characteristics of care home populations, with respect to fracture risk factors, have not been well-defined.

**Aim:**

To describe osteoporosis-related characteristics among care home residents, including fracture risk factors, fracture rates, post-fracture outcomes, and osteoporosis treatment duration.

**Design & setting:**

A descriptive cohort study of care home residents aged ≥60 years (*n* = 8366) and a matched cohort of non-care home residents (*n* = 16 143) in England from 2012 to 2019. Clinical Practice Research Datalink (CPRD) linked to Hospital Episode Statistics (HES) and Office for National Statistics (ONS) death data were used.

**Method:**

The characteristics were assessed using descriptive statistics. Fracture risk factors and fracture rates were described in both the care home and matched population. In the care home population, Kaplan–Meier curves were plotted to assess osteoporosis treatment duration.

**Results:**

At index, fracture risk factors were more common in care home residents versus the matched cohort, including body mass index (BMI) <18.5 (12.2% versus 5.1%), history of falls (48.9% versus 30.7%), prior fracture (26.5% versus 10.8%), and prior hip fracture (17.1% versus 5.8%). Fracture rate was 43.5 (95% confidence interval [CI] = 39.7 to 47.5) in care home residents and 28.0 (95% CI = 26.3 to 29.9) per 1000 person–years in the matched cohort. Overall, osteoporosis treatment was initiated in 3.6% (*n* = 225/6265) of care home residents and 45.9% remained on treatment at 12 months. Among care home residents who experienced fracture, 21.9% (*n* = 72/329) received an osteoporosis diagnosis; 21.2% (*n* = 63/297) initiated osteoporosis treatment post-hip fracture.

**Conclusion:**

Care home residents had more fracture risk factors and higher fracture rates than the matched cohort; however, osteoporosis diagnosis, treatment rates, and treatment duration were low. There is an opportunity to improve osteoporosis management in this vulnerable population.

## How this fits in

The characteristics of care home populations with respect to fracture risk factors have not been well-studied, and limited information is available from England. This study shows that care home residents have more fracture risk factors and experience higher fracture rates than the general population, but had low initiation and short duration of osteoporotic treatment. There is an opportunity for clinicians to reduce the gap in osteoporosis diagnosis and treatment.

## Introduction

In England, an estimated 418 710 people reside in care homes (3.4% of the population aged ≥65 years).^1^ Care homes include both residential (providing personal care to people with some independence but possibly not fully mobile and unable to live independently) and nursing homes (additionally offering on-site nursing care). Some care homes provide a mixture of residential and nursing care. Residents of care homes are likely to be frailer than the general population, and have more comorbidities and more complex medical needs.^2,3^

Osteoporosis prevalence increases with age,^4^ hence the burden of osteoporosis is high in the care home population. Many fracture risk factors are more common in care home populations than the general population; for example, low BMI, lack of physical activity, dementia, and conditions increasing fall risk including muscle weakness, balance problems, impaired vision, and stroke.^5–7^ Moreover, previous fracture is a risk factor for subsequent fracture,^8^ particularly hip fractures, which lead to nursing home admission in 17% of individuals.^9^

The care home population therefore represents a population at potentially high risk of fracture, who may benefit from targeted fracture-risk screening and prevention. However, the characteristics of care home populations, with respect to fracture risk and subsequent care, have not been well-defined in the UK.

This study aimed to describe osteoporosis-related characteristics among care home residents in England, including risk factors for osteoporotic fracture, fracture incidence, and post-fracture management and outcomes. To place the care home population and its fracture risk into context, the clinical characteristics and fracture rates of a matched non-care home population were also described.

## Method

### Study design and data sources

A descriptive cohort study was conducted of care home residents compared with a matched (age, sex, and practice) cohort of non-care home residents in England. Fracture risk factors, fracture rates, and patterns of care in care home residents were described. To place the care home population and its fracture risk into context, the clinical characteristics and fracture rates of a matched non-care home population were also described.

The study used primary care electronic health record data from the CPRD GOLD,^10^ linked hospital admissions data from HES,^11^ and linked Office for National Statistics (ONS) death data.^12^

The index date was the first date an individual was recorded as having care home residency during the cohort identification period (1 January 2012 to 31 December 2018). The follow-up period began at the index date and ended at the first of the following: record of residency outside care home; death; no longer registered with practice; GP practice no longer contributing to CPRD; or end of study period (31 December 2019).

### Study participants

Care home residents had to be aged ≥60 years at index date; resident within a care home during the cohort identification period (1 January 2012 to 31 December 2018); data had to be an acceptable quality; participants needed to be registered with a practice for at least 1 year before index date; and be eligible for HES and ONS linkage. Individuals with a care home residency record in the 24 months before the index date were excluded. Using the same inclusion and exclusion criteria, two non-care home residents for every care home resident were matched on year of birth, sex, and general practice, in calendar date order. Non-care home residents were assigned the same index date as their matched care home resident.

### Variables

Care home residency (nursing or residential care homes) was identified through primary care morbidity coding for specific place of residence.^13^ Individuals were assumed to remain in the care home unless otherwise indicated.

Demographic and clinical characteristics were identified using primary care records in the 24 months before the index date. Demographic characteristics included age, sex, and ethnic group (White, South Asian, Black, mixed, and other). Clinical characteristics included the following: BMI (calculated using height and weight measurements); smoking status (never, current, and ex); and alcohol use (current, ex, and never). Ethnic group, smoking status, and alcohol use were identified using morbidity coding.

All other fracture risk factors, including history of falls, history of fracture, comorbidities (osteoporosis, rheumatoid arthritis, diabetes mellitus, chronic kidney disease, chronic obstructive pulmonary disease, dementia, Parkinson’s disease, cardiovascular disease, cerebrovascular disease, and cancer), and use of osteoporosis medications and glucocorticoids were assessed at any point before the index date using primary care morbidity coding, prescriptions (see Supplementary Boxes S1 and S2), and International Classification of Diseases, version 10 (ICD-10) codes (fracture history) in HES data. Osteoporosis treatments included the following: bisphosphonates (oral and parenteral separately); denosumab; raloxifene; teriparatide; and strontium ranelate.

Post-index fractures were identified using ICD-10 codes recorded in hospital admissions (HES) data (see Supplementary Box S3) and categorised by the following type: hip; vertebral; non-hip non-vertebral; and any (hip, clinical vertebral, and non-hip non-vertebral). Fracture rates were calculated for each type of fracture, based on the initial fracture, that is, a fracture that occurred after the index date with no fracture at the same site in the 180 days before (that is, wash-out period). Transport accident-related fractures (recorded on the same day or within 7 days after the accident) were excluded to focus on osteoporotic fracture outcomes. Cumulative Incidence Competing Risk (CICR) estimates of fracture (any type and hip fracture) were also calculated to account for competing risk of death.

Among care home residents only, post-fracture management and outcomes were assessed including osteoporosis diagnosis and treatment initiation, length of hospitalisation, 1-year mortality, and osteoporosis treatment duration. Osteoporosis diagnosis and treatments were identified using primary care morbidity coding and prescriptions (see Supplementary Boxes S1 and S2) during the 12 months after the fracture. Duration of hospital stay in HES was used to determine length of hospitalisation. ONS death data were used to identify deaths.

Treatment duration was defined as time from osteoporosis treatment initiation to first treatment gap of ≥60 days. The gap was counted from the end of first prescription (based on days supplied) to date of subsequent prescription. If an individual received another prescription before the end of one prescription, then that end date was disregarded and a new end date was estimated for the subsequent prescription.

### Statistical methods

All statistical analyses were performed using SAS (version 9.4).

Demographic and clinical characteristics of the care home residents and matched cohort were summarised using descriptive statistics. Charlson Comorbidity Index was calculated using updated comorbidity weights.^14,15^

Crude fracture rates (per 1000 person–years) during follow-up and 95% CIs were calculated for care home residents and the matched cohort. CICR estimates of fracture (any type and hip fracture) and 95% CIs were calculated in care home and matched cohorts.^16^ In the care home population, the proportions of care home residents experiencing fracture who went on to receive a diagnosis of osteoporosis and initiated osteoporosis treatment in the 12 months post-fracture were calculated. Median length of first hospitalisation in the 90 days following fracture diagnosis and 1-year mortality were also calculated. All outcomes were assessed after initial fracture and by fracture type.

Treatment duration was assessed among care home residents initiating osteoporosis treatment after index date with no treatment record before the index date. Kaplan–Meier curves were used to visualise osteoporosis treatment duration in care home residents. Individuals were censored at death, no longer registered with the practice, or the practice was no longer contributing to CPRD. Percent of care home residents remaining on treatment and 95% CIs were reported at 1 year, 2 years, and 4 years after treatment initiation.

In subgroup analyses, the percent of care home residents remaining on treatment for those with and without a fracture within 12 months before treatment initiation was calculated.

## Results

**Figure 1.**
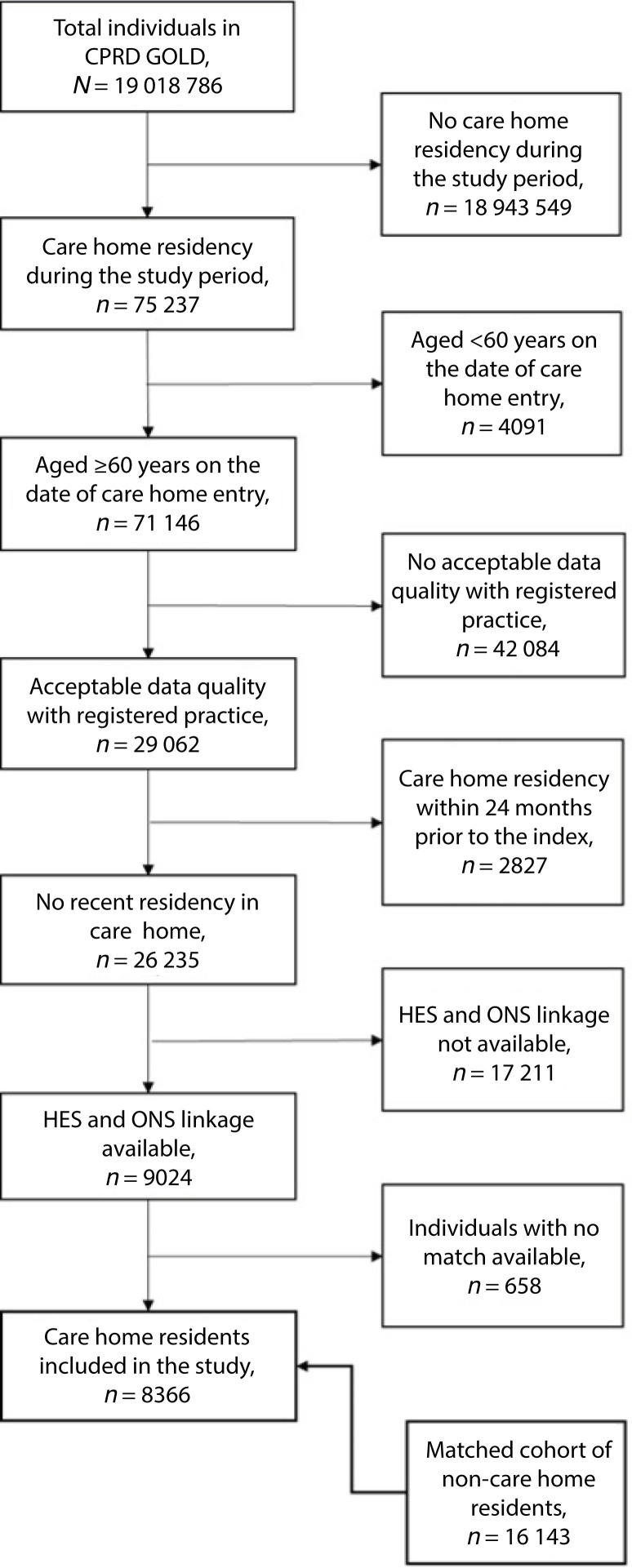
Study population flow diagram. Cohort matched on age, sex, and practice. CPRD = Clinical Practice Research Datalink. HES = Hospital Episode Statistics. ONS = Office for National Statistics.

A total of 8366 care home residents and 16 143 matched individuals were identified (Figure 1). Median follow-up for care home residents was 328 days (interquartile range [IQR] 133–674) and 603 days (IQR 292–1184) for the matched cohort.

In both care home resident and matched cohort, mean age was approximately 84 years and 69.8% were female (Table 1).

**Table 1.** Baseline characteristics of care home residents and matched non-care home cohort in England from 2012 to 2018

**Characteristic**	Care home residents, *n* (%)^a^	Non-care home residents, *n* (%)^a^
Total	8366	16 143
Age, years, mean±SD	84.6±8.3	84.3±8.2
Sex, female	5839 (69.8)	11 220 (69.5)
Ethnic group		
White	7968 (95.2)	14 486 (89.7)
South Asian	33 (0.4)	200 (1.2)
Black	53 (0.6)	97 (0.6)
Mixed	7 (0.1)	32 (0.2)
Other	57 (0.7)	81 (0.5)
Missing	248 (3.0)	1247 (7.7)
Body mass index, kg/m^2^, mean±SD	24.2±5.7	25.7±5.0
Underweight (<18.5)	1017 (12.2)	822 (5.1)
Normal weight (18.5–24.9)	3161 (37.8)	6344 (39.3)
Overweight (25.0–29.9)	1002 (12.0)	2709 (16.8)
Obese (>30.0)	1750 (20.9)	5192 (32.2)
Missing	1436 (17.2)	1076 (6.7)
Alcohol drinking status		
Current	3293 (39.4)	9774 (60.5)
Ex	862 (10.3)	809 (5.0)
Never	2677 (32.0)	4354 (27.0)
Missing	1534 (18.3)	1206 (7.5)
Smoking status		
Current	2995 (35.8)	7198 (44.6)
Ex	2420 (28.9)	5170 (32.0)
Never	2837 (33.9)	3608 (22.4)
Missing	114 (1.4)	167 (1.0)
History of falls	4090 (48.9)	4961 (30.7)
History of fracture		
Any	2221 (26.5)	1738 (10.8)
Hip	1430 (17.1)	934 (5.8)
Vertebral	209 (2.5)	135 (0.8)
Non-hip, non-vertebral	972 (11.6)	881 (5.5)
Comorbidities		
Osteoporosis	1724 (20.6)	2646 (16.4)
Rheumatoid arthritis	198 (2.4)	334 (2.1)
Diabetes mellitus	1612 (19.3)	2661 (16.5)
Chronic kidney disease	2846 (34.0)	5386 (33.4)
Chronic obstructive pulmonary disease	670 (8.0)	1174 (7.3)
Dementia	3887 (46.5)	1102 (6.8)
Parkinson’s disease	384 (4.6)	150 (0.9)
Cardiovascular disease^b^	3622 (43.3)	7132 (44.2)
Cerebrovascular disease^c^	2230 (26.7)	2226 (13.8)
Specific cancers^d^	1174 (14.0)	2247 (13.9)
Charlson Comorbidity Index, mean±SD	2.9±2.1	2.3±2.0
Anti-osteoporosis medications		
Any	2101 (25.1)	3369 (20.9)
Oral bisphosphonates	2005 (24.0)	3273 (20.3)
Parenteral bisphosphonates	67 (0.8)	158 (1.0)
Denosumab	12 (0.1)	25 (0.2)
Raloxifene	32 (0.4)	78 (0.5)
Teriparatide	4 (0.05)	3 (0.02)
Strontium ranelate	175 (2.1)	231 (1.4)
Glucocorticoids	2737 (32.7)	6429 (39.8)

^a^Unless otherwise stated. ^b^Cardiovascular disease includes diagnoses of myocardial infarction, heart failure, sudden cardiac death, stable angina, unstable angina, acute coronary syndrome, percutaneous coronary intervention, coronary artery bypass graft, coronary thrombolysis, and coronary heart disease not otherwise specified. ^c^Stroke and non-stroke cerebrovascular disease. ^d^Acute lymphoblastic leukaemia, acute myeloid leukaemia, bladder cancer, brain cancer, breast cancer, colorectal cancer, gastric cancer, head and neck cancer, lung cancer, melanoma, multiple myeloma, neuroendocrine carcinoma, non-Hodgkin lymphoma, oesophageal cancer, ovarian cancer, pancreatic cancer, prostate cancer, renal cancer, thyroid cancer, and uterine cancer.

Fracture risk factors were more common in care home residents than the matched cohort, with lower mean BMI (24.2, standard deviation [SD] 5.7 versus 25.7, SD 5.0), and a high proportion with history of falls (48.9% versus 30.7%), any fracture (26.5% versus 10.8%), and hip fracture (17.1% versus 5.8%). Care home residents, in contrast to the matched population, had higher proportions of osteoporosis diagnoses (20.6% versus 16.4%), prior osteoporosis medication use (25.1% versus 20.9%), dementia (46.5% versus 6.8%), stroke (26.7% versus 13.8%), and Parkinson’s disease (4.6% versus 0.9%).

There were no notable differences in fracture risk factors between nursing and residential care home residents (see Supplementary Table S1).

### Fracture rates

**Figure 2.**
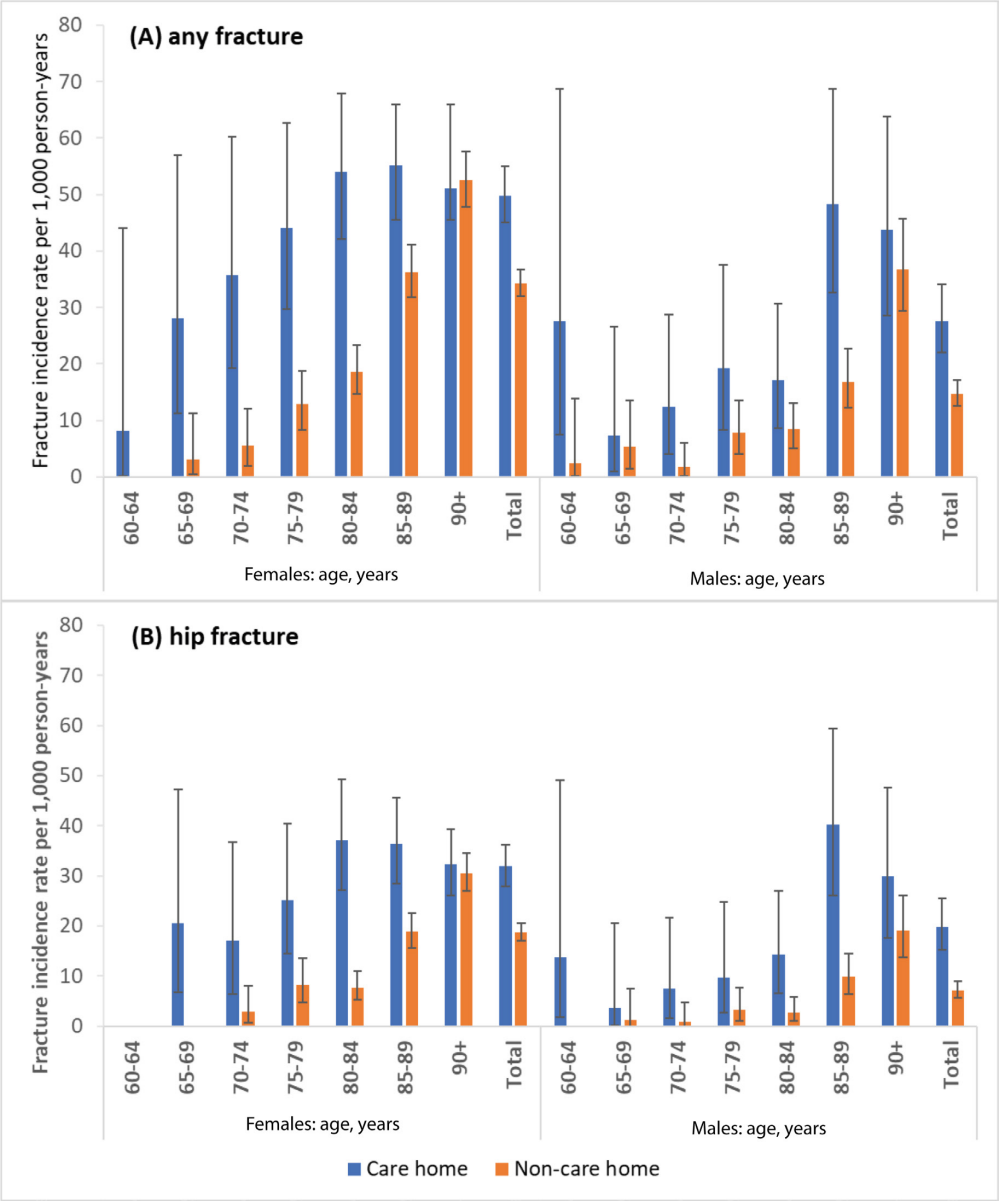
Crude fracture rates (error bars represent 95% confidence intervals) per 1000 person–years among care home residents and matched non-care home cohort in England from 2012 to 2019

Fracture rates were higher among care home residents than the matched cohort (Figure 2 and Supplementary Table S2). Overall fracture rates were 43.5 (95% CI = 39.7 to 47.5) per 1000 person–years in care home residents and 28.0 (95% CI = 26.3 to 29.9) per 1000 person–years in the matched cohort. Females had consistently higher fracture rates than males (females: care home residents 49.8 [95% CI = 45.0 to 55.0] per 1000 person–years, matched cohort 34.3 [95% CI = 32.0 to 36.8]; males: care home residents 27.6 [95% CI = 22.1 to 34.1] per 1000 person–years, matched cohort 14.7 [95% CI = 12.5 to 17.2]). Fracture rates increased with age in both cohorts and were higher among care home residents aged 70–89 years than in the matched cohort.

The higher fracture rates in the care home population were driven by hip fractures. Hip fracture rate in care home residents was 28.4 (95% CI = 25.3 to 31.8) per 1000 person–years, compared with 15.0 (95% CI = 13.7 to 16.3) per 1000 person–years in the matched cohort. The hip fracture rate in female care home residents (31.9 [95% CI = 28.0 to 36.2] per 1000 person–years) was higher than male care home residents (19.9 [95% CI = 15.2 to 25.5] per 1000 person–years). Rates of other fracture types were lower than hip fracture, and similar in both the care home resident and matched cohort (see Supplementary Table S2). There was no notable difference in fracture rates between nursing and residential care homes (see Supplementary Tables S3–S6).

The results of CICR analysis showed higher cumulative incidence of fracture for up to 2 years after the index date: incidence of any fracture after 18 months was 5.0% (95% CI = 4.5 to 5.5) in the care home populations and 3.6% (95% CI = 3.3 to 3.9) in the matched cohort; incidence of hip fracture was 3.8% (95% CI = 3.3 to 4.3) in the care home population and 2.7% (95% CI = 2.4 to 3.0) in the matched cohort (see Supplementary Tables S7 and S8). There was a notable reduction in the number of care home residents at risk for fracture over time, leading to similar CICR estimates in care home and matched populations at later time points.

### Care home residents: post-fracture management and outcomes

**Figure 3.**
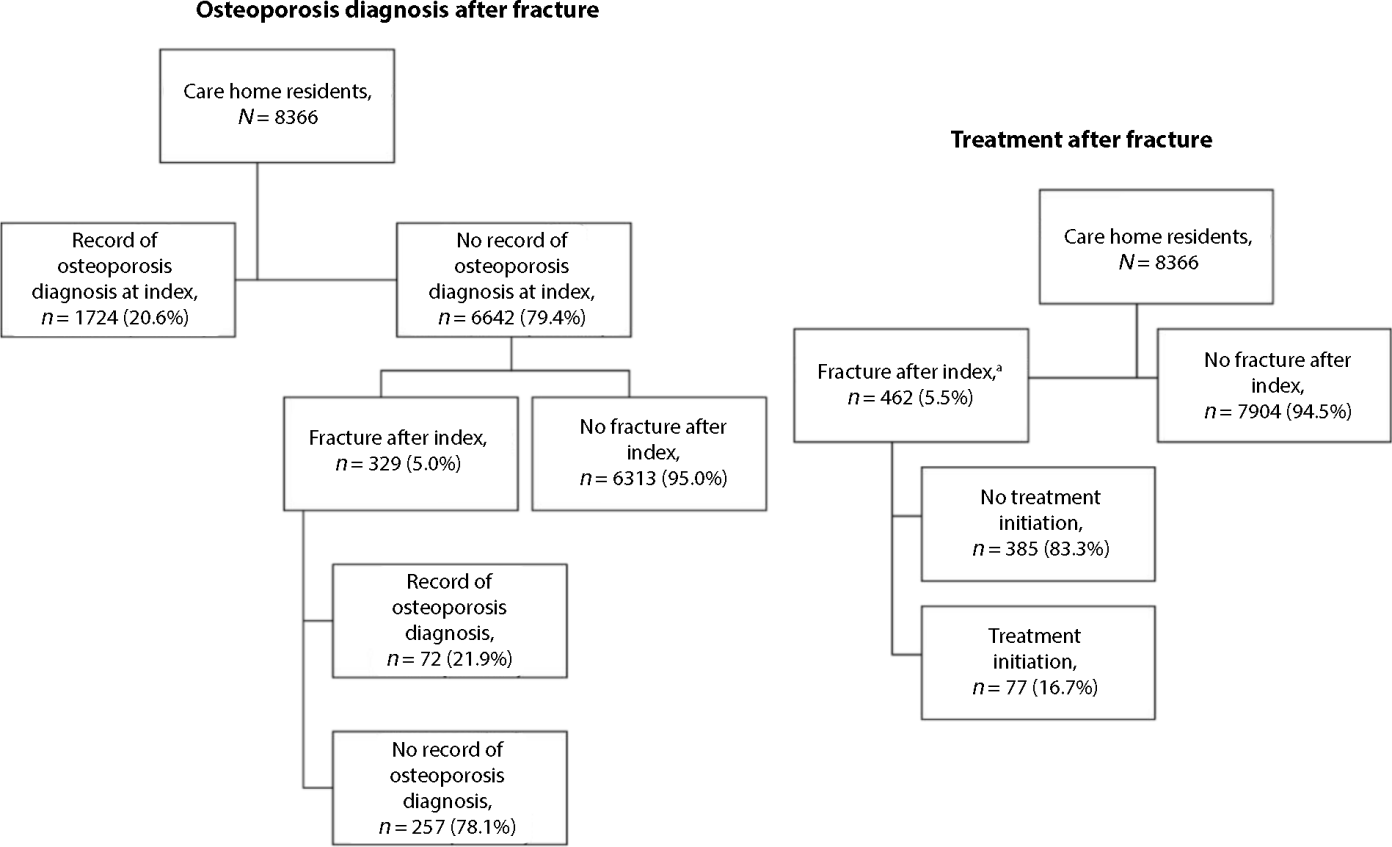
Osteoporosis diagnosis and treatment after fracture among care home residents in England from 2012 to 2019. ^a^Regardless of osteoporosis diagnosis.

The majority (79.4%, *n* = 6642/8366) of care home residents had no osteoporosis diagnosis at first record of care home residency. Of the care home residents with no osteoporosis diagnosis, 5.0% (*n* = 329/6642) experienced fracture during follow-up, 21.9% (*n* = 72/329) of whom had an osteoporosis diagnosis recorded post-fracture (Figure 3).

Overall, osteoporosis treatment was initiated in 16.7% (*n* = 77/462) of care home residents post-fracture. By fracture type, osteoporosis treatment was initiated in: 21.2% (*n* = 63/297) post-hip fracture; 4.0% (*n* = 2/50) post-vertebral fracture; and 11.0% (*n* = 14/127) post-non-hip non-vertebral fracture (data not shown).

Among care home residents, median hospital stay in the 90 days after fracture was 11 (IQR 6–22) days. Hip fractures resulted in a median hospital stay of 12 (IQR 8–22) days, vertebral fractures 8.5 (IQR 4–16) days, and non-hip non-vertebral 7.5 (IQR 1–23) days (data not shown).

Overall, 40.5% (*n* = 187/462) of care home residents died in the year post-fracture. One-year mortality was higher in males than females after any type of fracture (56.0% [95% CI = 44.7 to 66.8] versus 37.0% [95% CI = 32.2 to 41.1]), and after hip fracture (60.0% [95% CI = 46.5 to 72.4] versus 38.0% [95% CI = 31.8 to 44.5]). One-year mortality was similar for individuals living in residential and nursing care homes (see Supplementary Table S9).

### Care home residents: treatment duration

In total, 3.6% (*n* = 225/6265) of care home residents were new users of osteoporosis treatment after first care home residency record. Most (96.0%, *n* = 216/225) new users received oral bisphosphonates, with the remaining individuals receiving the following: parenteral bisphosphonates, 1.3% (*n* = 3/225); the RANKL inhibitor denosumab, 1.8% (*n* = 4/225); or strontium ranelate, 1.8% (*n* = 4/225) (data not shown).

**Figure 4.**
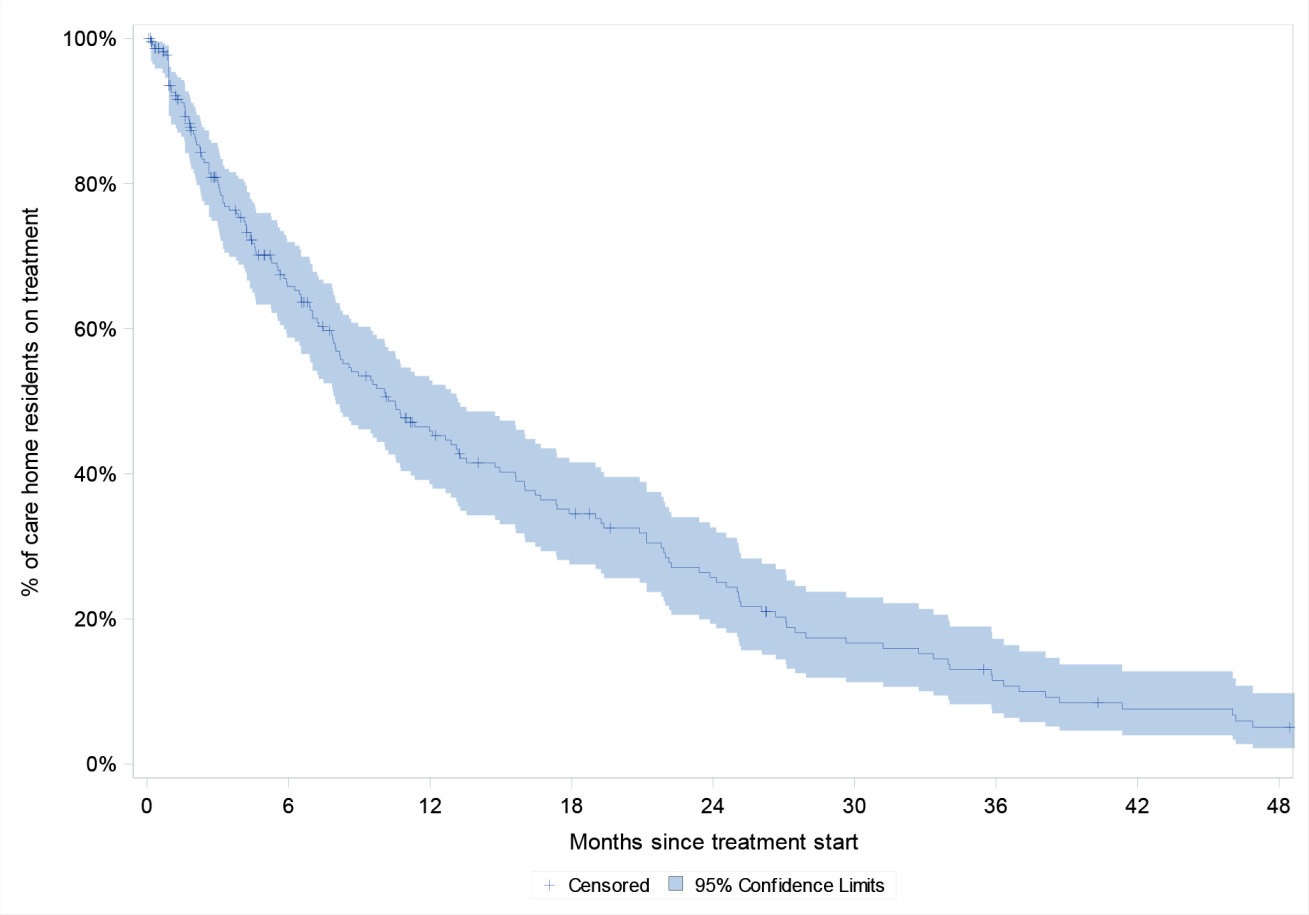
Kaplan–Meier curve of treatment duration among new users of osteoporosis treatment in care homes in England from 2012 to 2019

The proportion of care home residents who persisted with osteoporosis treatment 12 months after treatment initiation was 45.9% (95% CI = 38.6 to 52.9), dropping to 25.7% (95% CI = 19.3 to 32.6) after 24 months and 5.1% (95% CI = 2.2 to 9.8) after 48 months (Figure 4). Median osteoporosis treatment duration was 10.5 ( IQR 4.1–24.6) months. Treatment duration was similar among care home residents who did and did not experience fracture within 12 months before treatment initiation (see Supplementary Table S10).

## Discussion

### Summary

This study found that fracture risk factors,^8^ such as low BMI, prior fracture, prior hip fracture, and history of falls, were more common among care home residents than in a matched cohort of non-care home residents. In addition, fracture rates were higher in the care home population, predominantly owing to an increased rate of hip fracture. Post-fracture osteoporosis diagnosis and treatment rates were low, with short duration of osteoporosis treatment.

### Strengths and limitations

CPRD is a well-established longitudinal database representative of the UK population.^10^ Moreover, CPRD level of completeness of residency recording has been found to be comparable with English census data.^13^ The present study demonstrates that CPRD can be used to assess clinical outcomes in care home populations.

The main analysis used crude fracture rates per 1000 person–years to show that rates of fracture were higher in the care home population, which was mainly driven by increased hip fracture. In older populations with substantial comorbidities, estimations of clinical outcomes can be overestimated owing to high mortality rates or differences in mortality rates between groups.^16^ In the present study, the length of follow-up in care homes was about half that of the control group, suggesting a discrepancy. To account for this potential bias, the cumulative incidence of any fracture and hip fracture was estimated with death as a competing risk. The cumulative incidence of fracture was higher in the care home group for up to 2 years, after which time only 10% of the care home population remained in the analysis and CIs around the point estimates began to widen. The results indicate fracture rates in the care home population are higher than matched counterparts even when their increased risk of mortality is taken into account.

Morbidity codes were used to identify care home residency and assign an index date. The date of first coded care home residency recording is unlikely to reflect the precise date of care home entry, it is more likely to capture the first GP interaction during care home residency. It is likely that the authors only captured clinical vertebral fractures as most vertebral fractures are asymptomatic and remain undiagnosed. Only treatments prescribed in primary care are captured in CPRD; therefore, treatments administered in secondary care may not be captured. However, both oral bisphosphonates and RANKL inhibitor denosumab can be prescribed by GPs (although denosumab is more likely to be initiated in secondary care) so it is unlikely that osteoporosis treatment rates were underestimated.

### Comparison with existing literature

The findings are consistent with a previous study reporting more chronic diseases, including cerebrovascular disease and dementia, in care home residents in the UK primary care population.^7^ Consistent with the same study, the proportion of care home residents in the present study with comorbidities (including cardiovascular disease, cancer, diabetes, and chronic obstructive pulmonary disease) was similar between nursing and residential care populations. A higher prevalence of Parkinson’s disease was found in care home residents than non-care home residents, with a similar proportion of non-care home residents with Parkinson’s disease to that reported in CPRD.^17,18^

Consistent with increased fracture risk factors, higher fracture rates were found among care home residents compared with the matched non-care home cohort. Reassuringly, hip fracture rates in the present study were similar to those among US nursing facility residents.^19^

The National Institute for Health and Care Excellence (NICE) guidelines recommend oral bisphosphonates as a first-line treatment for osteoporosis.^20^ Consistent with these guidelines, most care home residents receiving osteoporosis therapy in the present study were treated with oral bisphosphonates. However, oral bisphosphonates are known to have gastrointestinal tolerability issues, which may reduce uptake among care home residents, who have a wide range of comorbidities and who are prescribed multiple other medications.^21^ Short duration of osteoporosis treatment was also observed (45.9% on treatment at 12 months), which is consistent with that previously reported in the general CPRD population.^22^

### Implications for practice and research

There are well-established risk factors for osteoporotic fracture,^23–28^ many of which are used in the Fracture Risk Assessment Tool (FRAX) algorithm.^29–31^ Compared with non-care home residents, it was found that care home residents were more likely to have several FRAX risk factors, including lower BMI, history of falls, and prior fracture, indicating care home residents are a high-fracture-risk population.

The findings of higher fracture rates in the care home population indicate that, while care homes can provide a physically safer environment and closer monitoring of health and nutrition, this may not be sufficient to overcome the numerous comorbidities in the population that translate to high fracture risk.

The increased fracture rate that was observed in the study was largely driven by hip fracture, the most clinically serious fracture, carrying the highest burden for individuals and healthcare systems.^32–34^ It was found that median initial hospital stay duration among care home residents with hip fracture was 12.0 days (with lower median stays of 8.5 days for vertebral and 7.5 days for non-hip non-vertebral fractures) indicating that care home residency does not eliminate the burden of hospital care in cases of clinically meaningful fractures.

While randomised controlled trials and real-world evidence show osteoporosis treatments are effective in reducing fracture incidence,^35,36^ only 16.7% of care home residents in the present study received osteoporosis treatment post-fracture. High prevalence of chronic kidney disease (contraindication for the oral bisphosphonate use) in the study population could contribute to low overall osteoporosis treatment rates. A large treatment gap in osteoporosis has been previously reported,^37,38^ it is particularly striking in the study's older, post-fracture population. This study further highlights an opportunity for improved treatment choices in individuals with multiple osteoporosis and fracture risk factors. It was found only 21.9% of care home residents had a diagnosis of osteoporosis recorded following a fracture. Low osteoporosis diagnosis rates are associated with low treatment rates and contribute to the osteoporosis treatment gap in primary care.^39,40^

In conclusion, it was found that older care home residents in England had more fractures, particularly hip fractures, than older people in the community. While a large proportion of the care home population are at high risk of fracture, a large osteoporosis treatment gap remains, and the opportunity exists to improve screening, diagnosis, and management of osteoporosis in this vulnerable population.

## Data

Data are available on request from the CPRD and is subject to the protocol approval via CPRD’s Research Data Governance process: https://cprd.com/data-access

## References

[1] Office for National Statistics (2020). Care home and non-care home populations used in the deaths involving COVID-19 in the care sector article, England and Wales. www.ons.gov.uk/peoplepopulationandcommunity/birthsdeathsandmarriages/deaths/adhocs/12215carehomeandnoncarehomepopulationsusedinthedeathsinvolvingcovid19inthecaresectorarticleenglandandwales.

[2] Barker RO, Hanratty B, Kingston A (2021). Changes in health and functioning of care home residents over two decades: what can we learn from population-based studies?. Age Ageing.

[3] Gordon AL, Franklin M, Bradshaw L (2014). Health status of UK care home residents: a cohort study. Age Ageing.

[4] Borgström F, Karlsson L, Ortsäter G (2020). Fragility fractures in Europe: burden, management and opportunities. Arch Osteoporos.

[5] Chen JS, Sambrook PN, Simpson JM (2009). Risk factors for hip fracture among institutionalised older people. Age Ageing.

[6] Shah SM, Carey IM, Harris T (2011). Quality of chronic disease care for older people in care homes and the community in a primary care pay for performance system: retrospective study. BMJ.

[7] Shah SM, Carey IM, Harris T (2010). Identifying the clinical characteristics of older people living in care homes using a novel approach in a primary care database. Age Ageing.

[8] Kanis JA, Johnell O, Oden A (2008). FRAX and the assessment of fracture probability in men and women from the UK. Osteoporos Int.

[9] Osnes EK, Lofthus CM, Meyer HE (2004). Consequences of hip fracture on activities of daily life and residential needs. Osteoporos Int.

[10] Herrett E, Gallagher AM, Bhaskaran K (2015). Data resource profile: Clinical Practice Research Datalink (CPRD). Int J Epidemiol.

[11] NHS Digital (2023). Hospital Episode Statistics (HES). digital.nhs.uk/data-and-information/data-tools-and-services/data-services/hospital-episode-statistics.

[12] Office for National Statistics Deaths. www.ons.gov.uk/peoplepopulationandcommunity/birthsdeathsandmarriages/deaths.

[13] Jain A, van Hoek AJ, Walker JL (2017). Identifying social factors amongst older individuals in linked electronic health records: an assessment in a population based study. PLoS One.

[14] Metcalfe D, Masters J, Delmestri A (2019). Coding algorithms for defining Charlson and Elixhauser co-morbidities in Read-coded databases. BMC Med Res Methodol.

[15] Quan H, Li B, Couris CM (2011). Updating and validating the Charlson Comorbidity Index and score for risk adjustment in hospital discharge abstracts using data from 6 countries. Am J Epidemiol.

[16] Berry SD, Ngo L, Samelson EJ, Kiel DP (2010). Competing risk of death: an important consideration in studies of older adults. J Am Geriatr Soc.

[17] Office for National Statistics (2021). Population estimates for the UK, England and Wales, Scotland and Northern Ireland: mid-2020. www.ons.gov.uk/peoplepopulationandcommunity/populationandmigration/populationestimates/bulletins/annualmidyearpopulationestimates/mid2020.

[18] Parkinson’s UK (2018). The incidence and prevalence of Parkinson’s in the UK. Results from the Clinical Practice Research Datalink summary report. www.parkinsons.org.uk/sites/default/files/2018-01/CS2960%20Incidence%20and%20prevalence%20report%20branding%20summary%20report.pdf.

[19] Berry SD, Lee Y, Zullo AR (2016). Incidence of hip fracture in U.S nursing homes. J Gerontol A Biol Sci Med Sci.

[20] National Osteoporosis Guideline Group (2021). Clinical guideline for the prevention and treatment of osteoporosis. https://www.nogg.org.uk.

[21] Chodick G, Moser SS, Goldshtein I (2016). Non-adherence with bisphosphonates among patients with osteoporosis: impact on fracture risk and healthcare cost‏. Expert Rev Pharmacoecon Outcomes Res.

[22] Morley J, Moayyeri A, Ali L (2020). Persistence and compliance with osteoporosis therapies among postmenopausal women in the UK Clinical Practice Research Datalink. Osteoporos Int.

[23] Balasubramanian A, Zhang J, Chen L (2019). Risk of subsequent fracture after prior fracture among older women. Osteoporos Int.

[24] Curtis EM, van der Velde R, Moon RJ (2016). Epidemiology of fractures in the United Kingdom 1988–2012: variation with age, sex, geography, ethnicity and socioeconomic status. Bone.

[25] Johnell O, Kanis JA, Oden A (2005). Predictive value of BMD for hip and other fractures. J Bone Miner Res.

[26] Kanis JA, Johnell O, Oden A (2001). Ten year probabilities of osteoporotic fractures according to BMD and diagnostic thresholds. Osteoporos Int.

[27] Klotzbuecher CM, Ross PD, Landsman PB (2000). Patients with prior fractures have an increased risk of future fractures: a summary of the literature and statistical synthesis. J Bone Miner Res.

[28] Parthan A, Kruse M, Yurgin N (2013). Cost effectiveness of denosumab versus oral bisphosphonates for postmenopausal osteoporosis in the US. Appl Health Econ Health Policy.

[29] Kanis JA, McCloskey EV, Johansson H (2010). Development and use of FRAX in osteoporosis. Osteoporos Int.

[30] Kanis JA, Oden A, Johansson H (2009). FRAX and its applications to clinical practice. Bone.

[31] University of Sheffield Fracture Risk Assessment Tool. https://frax.shef.ac.uk/FRAX/tool.aspx.

[32] Downey C, Kelly M, Quinlan JF (2019). Changing trends in the mortality rate at 1-year post hip fracture—a systematic review. World J Orthop.

[33] Johnell O, Kanis JA (2004). An estimate of the worldwide prevalence, mortality and disability associated with hip fracture. Osteoporos Int.

[34] Leal J, Gray AM, Prieto-Alhambra D (2016). Impact of hip fracture on hospital care costs: a population-based study. Osteoporos Int.

[35] Albert SG, Wood E (2021). Meta-analysis of clinical fracture risk reduction of antiosteoporosis drugs: direct and indirect comparisons and meta-regressions. Endocr Pract.

[36] Yusuf AA, Cummings SR, Watts NB (2018). Real-world effectiveness of osteoporosis therapies for fracture reduction in post-menopausal women. Arch Osteoporos.

[37] Harvey NCW, McCloskey EV, Mitchell PJ (2017). Mind the (treatment) gap: a global perspective on current and future strategies for prevention of fragility fractures. Osteoporos Int.

[38] Kanis JA, Norton N, Harvey NC (2021). Scope 2021: a new scorecard for osteoporosis in Europe. Arch Osteoporos.

[39] McCloskey E, Rathi J, Heijmans S (2021). The osteoporosis treatment gap in patients at risk of fracture in European primary care: a multi-country cross-sectional observational study. Osteoporos Int.

[40] McCloskey E, Rathi J, Heijmans S (2022). Prevalence of FRAX risk factors and the osteoporosis treatment gap among women ≥ 70 years of age in routine primary care across 8 countries in Europe. Arch Osteoporos.

